# Supplementary material for: Multiple structures of RNA polymerase II isolated from human nuclei by ChIP-CryoEM analysis
Source: Nat Commun. 2025 May 28;16:4724. doi: 10.1038/s41467-025-59580-x (PMC12119854; doi:10.1038/s41467-025-59580-x)
Supplement: Supplementary file 1 — Supplementary Information [file 41467_2025_59580_MOESM1_ESM.pdf]

Supplementary Information for:

Multiple structures of RNA polymerase II isolated from human nuclei by ChIP-CryoEM analysis

Tomoya Kujirai[1,2], Junko Kato[1], Kyoka Yamamoto[1,3], Seiya Hirai[1,3], Takeru Fujii[4], Kazumitsu Maehara[4], Akihito Harada[4,5], Lumi Negishi[1], Mitsuo Ogasawara[1], Yuki Yamaguchi[6], Yasuyuki Ohkawa[4], Yoshimasa Takizawa[1], Hitoshi Kurumizaka[1,2,3]*

[1]Laboratory of Chromatin Structure and Function, Institute for Quantitative Biosciences, The University of Tokyo, 1-1-1 Yayoi, Bunkyo-ku, Tokyo 113-0032, Japan.

[2]Laboratory for Transcription Structural Biology, RIKEN Center for Biosystems Dynamics Research, 1-7-22 Suehiro-cho, Tsurumi-ku, Yokohama 230-0045, Japan.

[3]Department of Biological Sciences, Graduate School of Science, The University of Tokyo, 1-1-1 Yayoi, Bunkyo-ku, Tokyo 113-0032, Japan.

[4]Division of Transcriptomics, Medical Institute of Bioregulation, Kyushu University, 3-1-1 Maidashi, Higashi, Fukuoka 812-0054, Japan.

[5]Department of Multi-Omics, Graduate School of Medical Sciences, Kyushu University, 3-1-1 Maidashi, Higashi-ku, Fukuoka 812-0054, Japan

[6]School of Life Science and Technology, Institute of Science Tokyo, 4259 Nagatsuta, Yokohama 226-8501, Japan.

*Corresponding author. Email: kurumizaka@iqb.u-tokyo.ac.jp

Supplementary information includes:
Supplementary Fig. 1–12
Source Data of Supplementary Fig. 1
Supplementary Data 1. The RNAPII-associated proteins identified by LC-MS/MS analysis (full list)

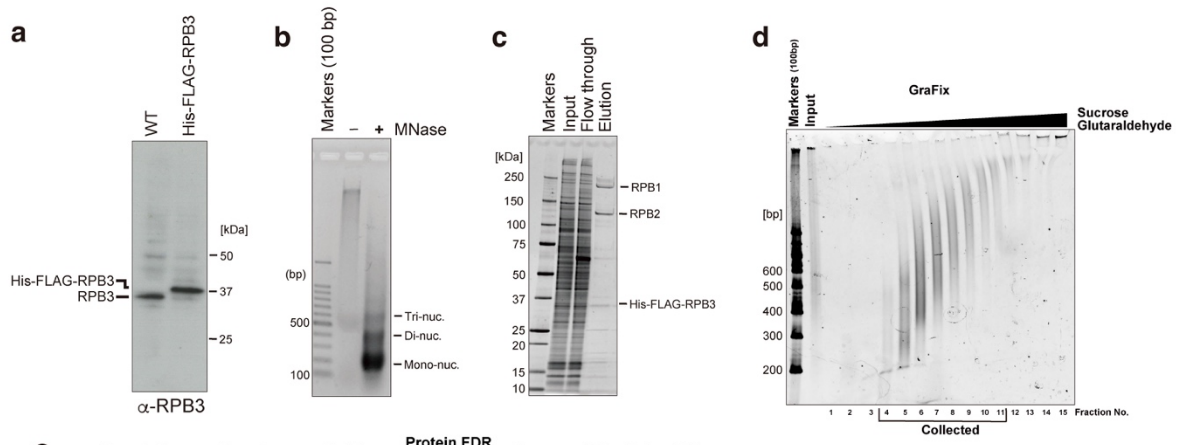

e

Description	Complex	Protein	Protein FDR confidence	Coverage [%]	Uniprot ID
	RNAPII	RPB1	High	79	P24928
		RPB2	High	84	P30876
		RPB3	High	94	P19387
Preinitiation complex	Mediator	MED1	High	52	Q15648
		MED14	High	59	O60244
		MED17	High	68	Q9NVC6
	TFIIH	XPB	High	39	P19447
		XPD	High	46	P18074
		p62	High	46	P32780
	TFIID	TAF4	High	31	O00268
		TAF5	High	35	Q15542
		TAF6	High	50	P49848
Elongation complex		TFIIS	High	29	P23193
	DSIF	SPT4	High	21	P63272
		SPT5	High	67	O00267
		SPT6	High	70	Q7KZ85
		IWS1	High	48	Q96ST2
		ELOF1	Medium	22	P60002
	PAF1C	PAF1	High	44	Q8N7H5
		CDC73	High	47	Q6P1J9
		CTR9	High	19	Q6PD62
	FACT	SPT16	High	66	Q9Y5B9
		SSRP1	High	54	Q08945
Chromatin	Nucleosome	H2A	High	78	Q6FI13
		H2B	High	84	Q99879
		H3	High	42	P84243
		H4	High	60	P62805
DNA-free RNAPII binding		RPAP2	High	59	Q8IXW5

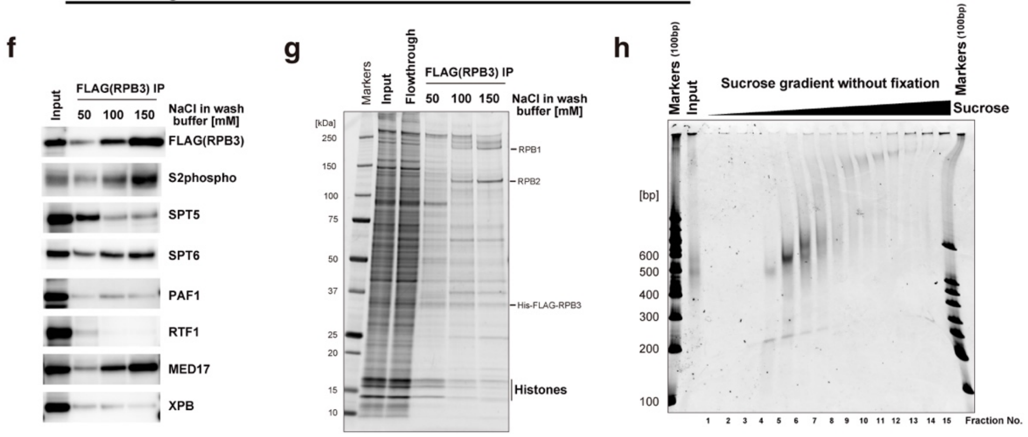

Supplementary Fig. 1. Preparation of RNAPII complexes from HeLa nuclei by ChIP-cryoEM. (a) Western blot of the wild-type and His-FLAG-expressing HeLa cells detected by an anti-RPB3 antibody. This experiment was performed once. **(b)** Chromatin digestion by micrococcal nuclease (MNase). The sonicated chromatin with/without MNase was analyzed by agarose gel electrophoresis with ethidium bromide staining. The bands corresponding to mono-, di-, and tri-nucleosomes are indicated. This experiment was repeated more than three times with similar results. **(c)** Purification of FLAG-tagged RNAPII by anti-FLAG antibody beads. This experiment was repeated more than three times with similar results. **(d)** Native polyacrylamide gel electrophoresis (PAGE) of the samples fractionated by GraFix. The nucleic acids (DNA and/or RNA) were visualized by SYBR Gold staining. This experiment

was repeated more than three times with similar results. **(e)** List of selected RNAPII-associated proteins identified by LC-MS/MS analysis. The full list of identified proteins is shown in Supplementary Data 1. This experiment was repeated twice with similar results. **(f)** Western blot of RNAPII-associated proteins. The indicated proteins were detected by antibodies. Different concentrations of NaCl were used to purify the proteins. Uncropped blots in Source Data. This experiment was repeated twice with similar results. **(g)** SDS-PAGE of the eluted proteins. The proteins were visualized by Oriole staining. This experiment was repeated twice with similar results. **(h)** Native-PAGE of the samples fractionated by sucrose gradient without fixation. The nucleic acids (DNA and/or RNA) contained in the fractionated samples were detected by SYBR Gold staining. This experiment was repeated twice with similar results.

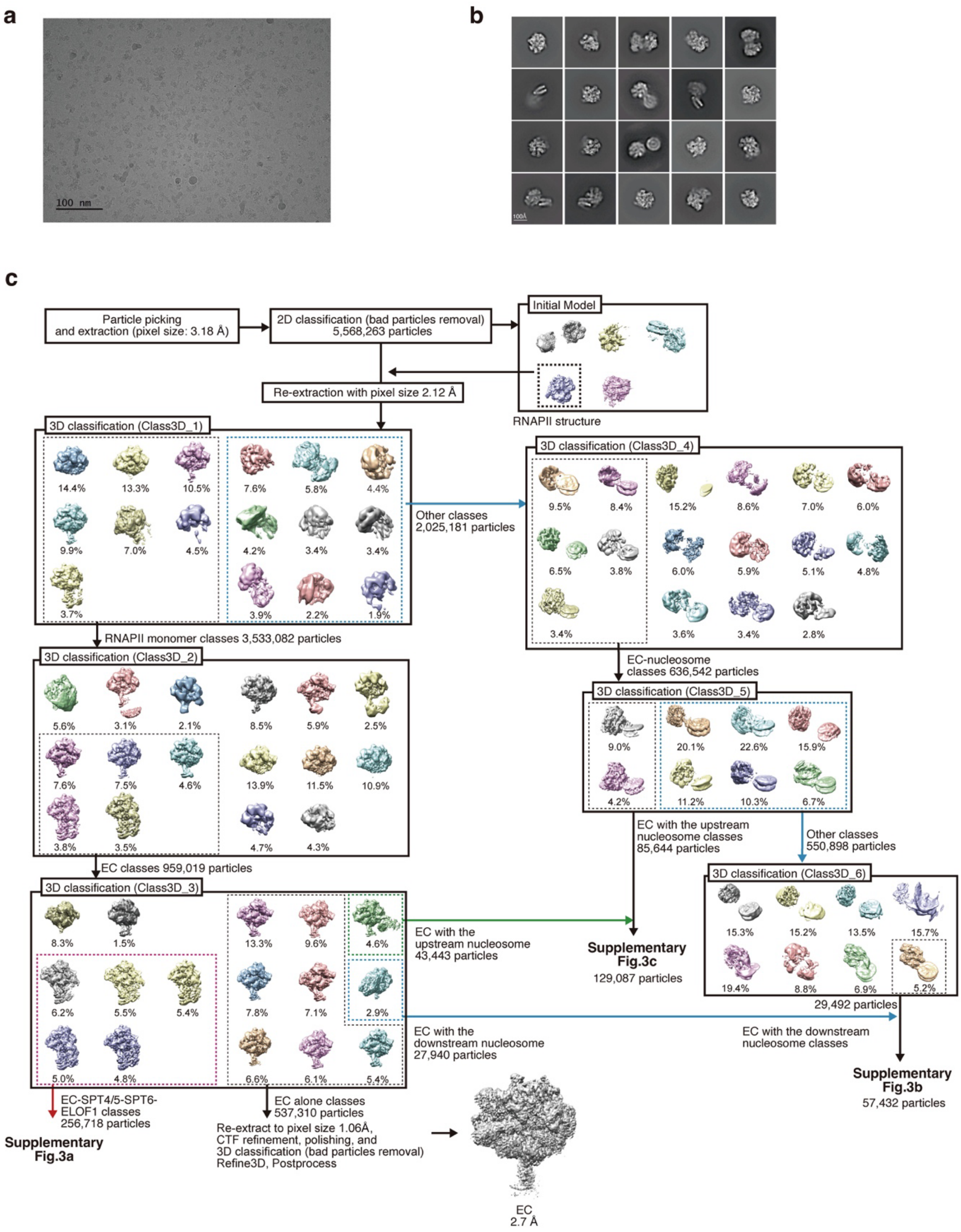

Supplementary Fig. 2. Cryo-EM data collection and image processing of RNAPII complexes extracted from HeLa nuclei. (a) Representative micrograph of the RNAPII complexes. The number of micrographs collected is shown in Table 1. **(b)** Representative 2D classes of the RNAPII complexes. The box size is 382 Å x 382 Å. **(c)** Workflow of the image processing.

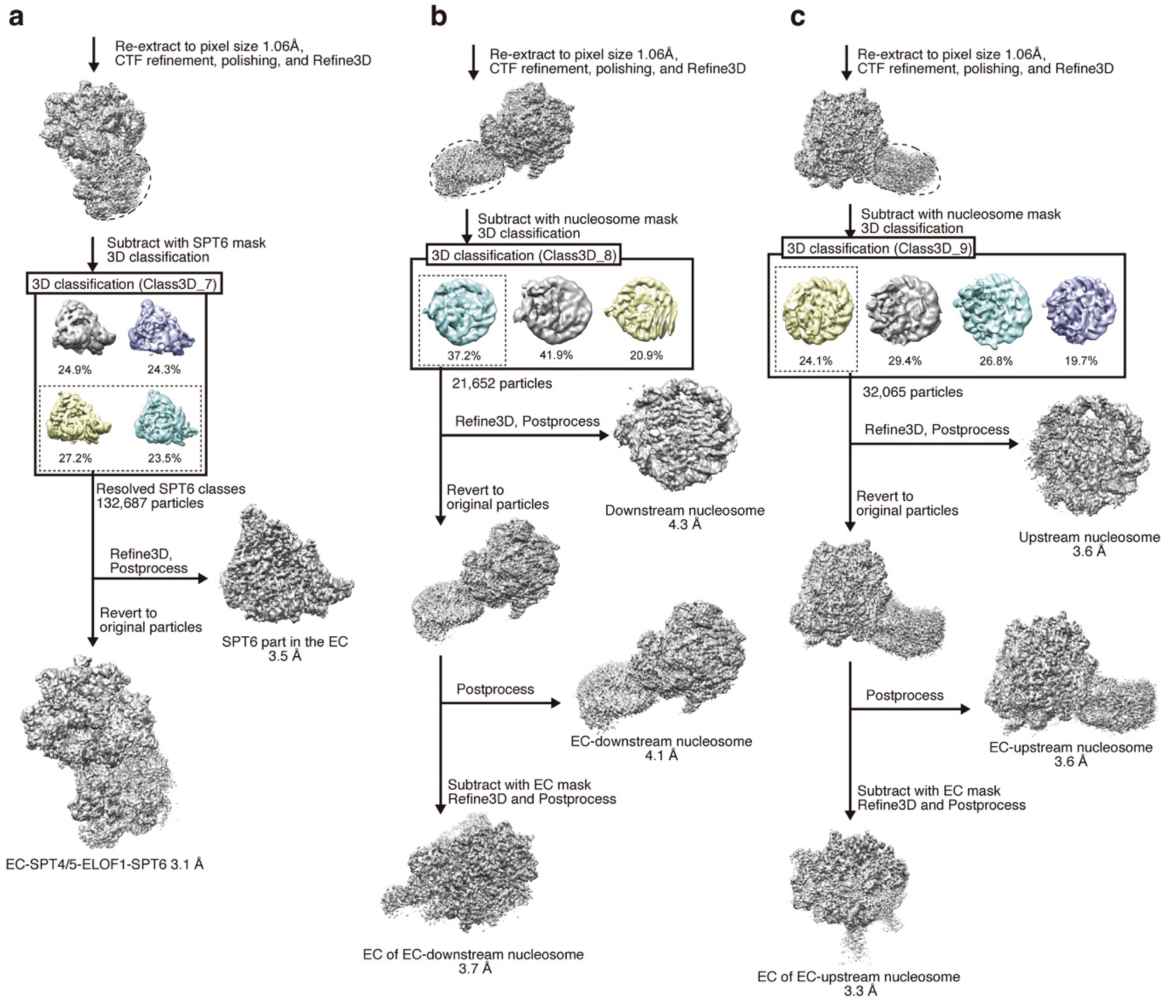

Supplementary Fig. 3. Image processing of the EC-SPT4/5-ELOF1-SPT6 and EC-nucleosome complexes. (a-c) Image processing of EC-SPT4/5-ELOF1-SPT6 (a), EC-downstream nucleosome (b), and EC-upstream nucleosome (c).

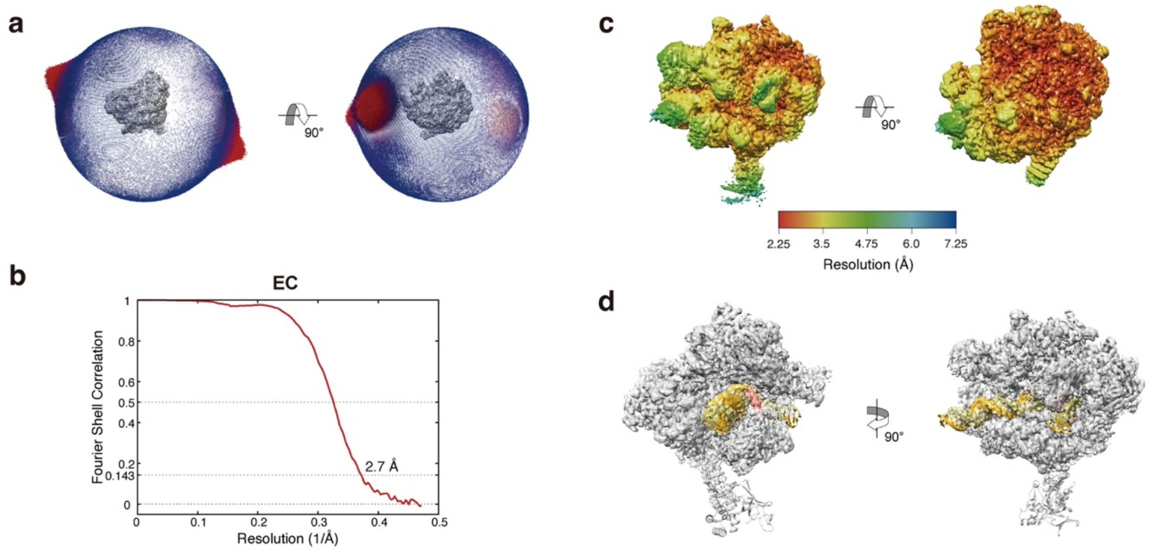

Supplementary Fig. 4. Quality of the EC structure. (a) Euler angular distribution of the structures of EC. **(b)** Fourier Shell Correlation (FSC) curves for EC. The resolution of the EC structure was estimated to be 2.7 Å by an FSC = 0.143. **(c)** Local resolution map of the EC structure. **(d)** Cryo-EM density of EC with the fitted atomic model.

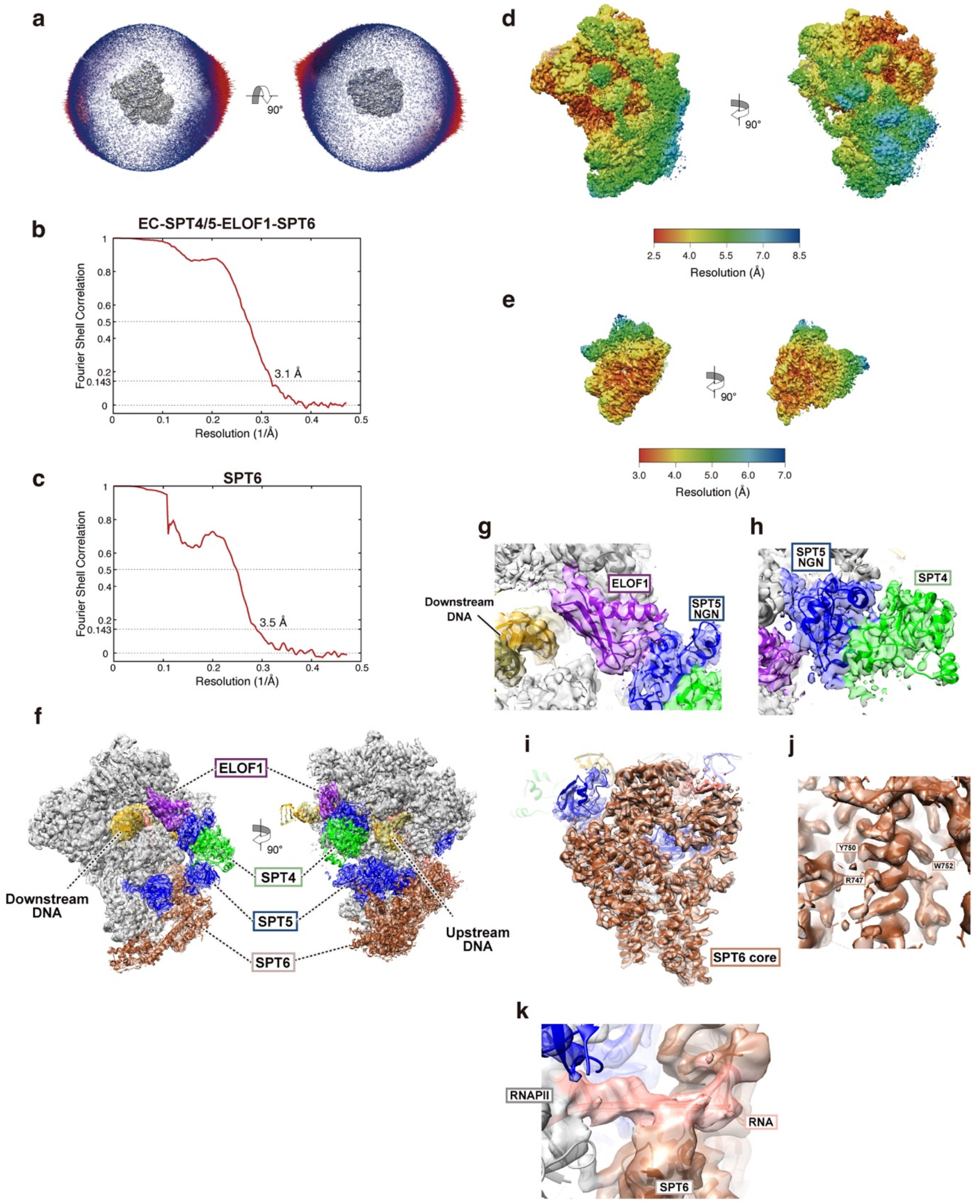

Supplementary Fig. 5. Quality of the EC-SPT4/5-ELOF1-SPT6 structure. (a) Euler angular distribution of the structure. **(b, c)** Fourier Shell Correlation (FSC) curves for EC **(b)** and SPT6 **(c)**. The resolutions were estimated to be 3.1 and 3.5 Å, respectively, by an FSC = 0.143. **(d, e)** Local resolution maps of the EC **(d)** and SPT6 **(e)** structures. **(f)** Cryo-EM density of EC-SPT4/5-ELOF1-SPT6 with the fitted atomic model. **(g, h)** Close-up views of the ELOF1 **(g)** and SPT5 NGN-SPT4 **(h)** densities with the fitted atomic models. **(i)** Cryo-EM density of SPT6 with the fitted atomic model. The focused refined map of SPT6 is shown. **(j)** Close-up of the cryo-EM density of SPT6. **(k)** RNA density around SPT6. The low pass filtered map at 5 Å is shown with the atomic models.

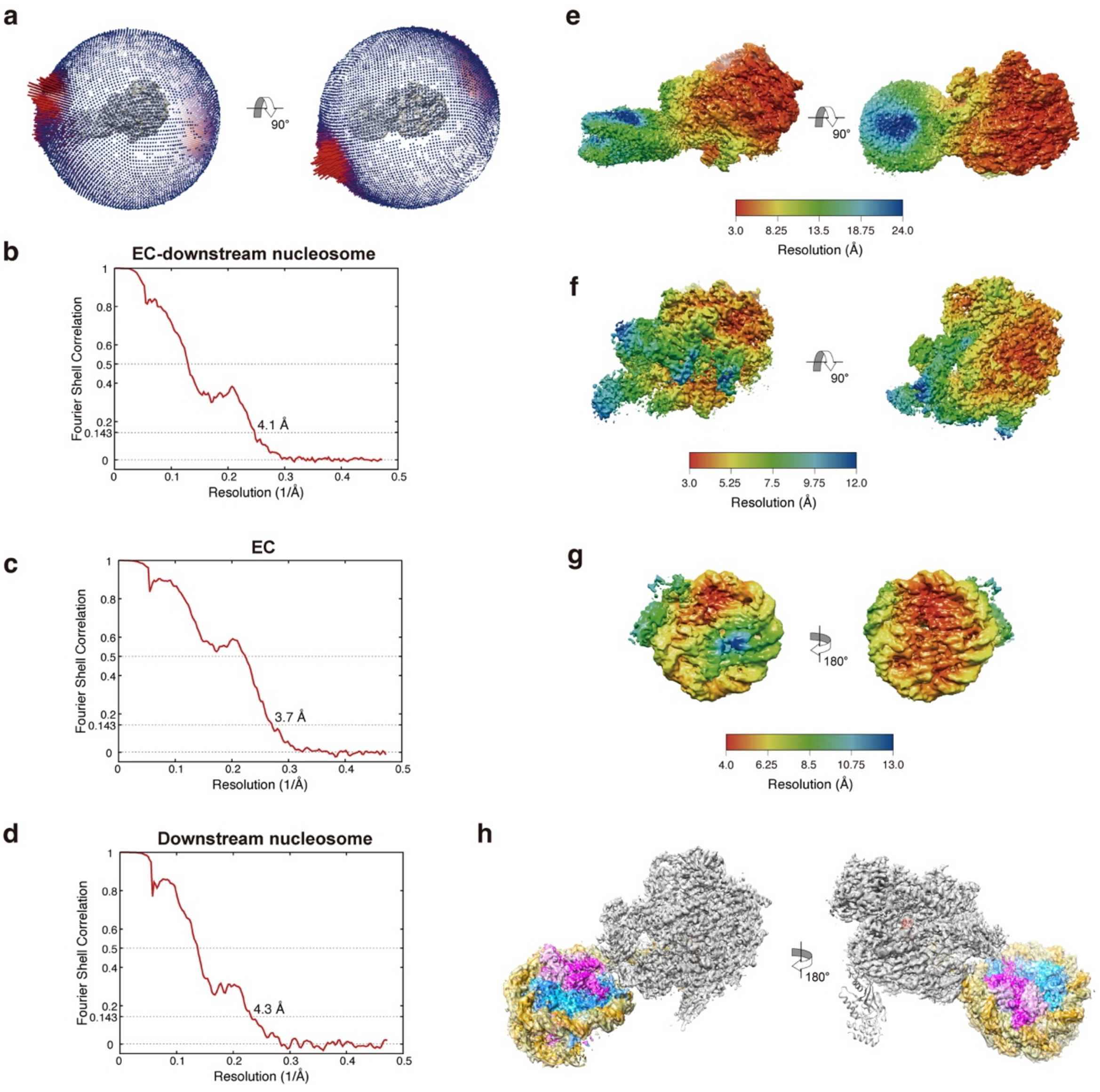

Supplementary Fig. 6. Quality of the EC-downstream nucleosome structure. (a) Euler angular distribution of the structure. **(b-d)** Fourier Shell Correlation (FSC) curves for the EC-downstream nucleosome (b), EC (c), and downstream nucleosome (d). The resolutions of the EC-downstream nucleosome, EC, and downstream nucleosome structures were estimated to be 4.1, 3.7, and 4.3 Å, respectively, by an FSC = 0.143. **(e-g)** Local resolution maps of the EC-downstream nucleosome (e), EC (f), and downstream nucleosome (g). **(h)** Cryo-EM density of the EC-downstream nucleosome composite map with the fitted atomic model.

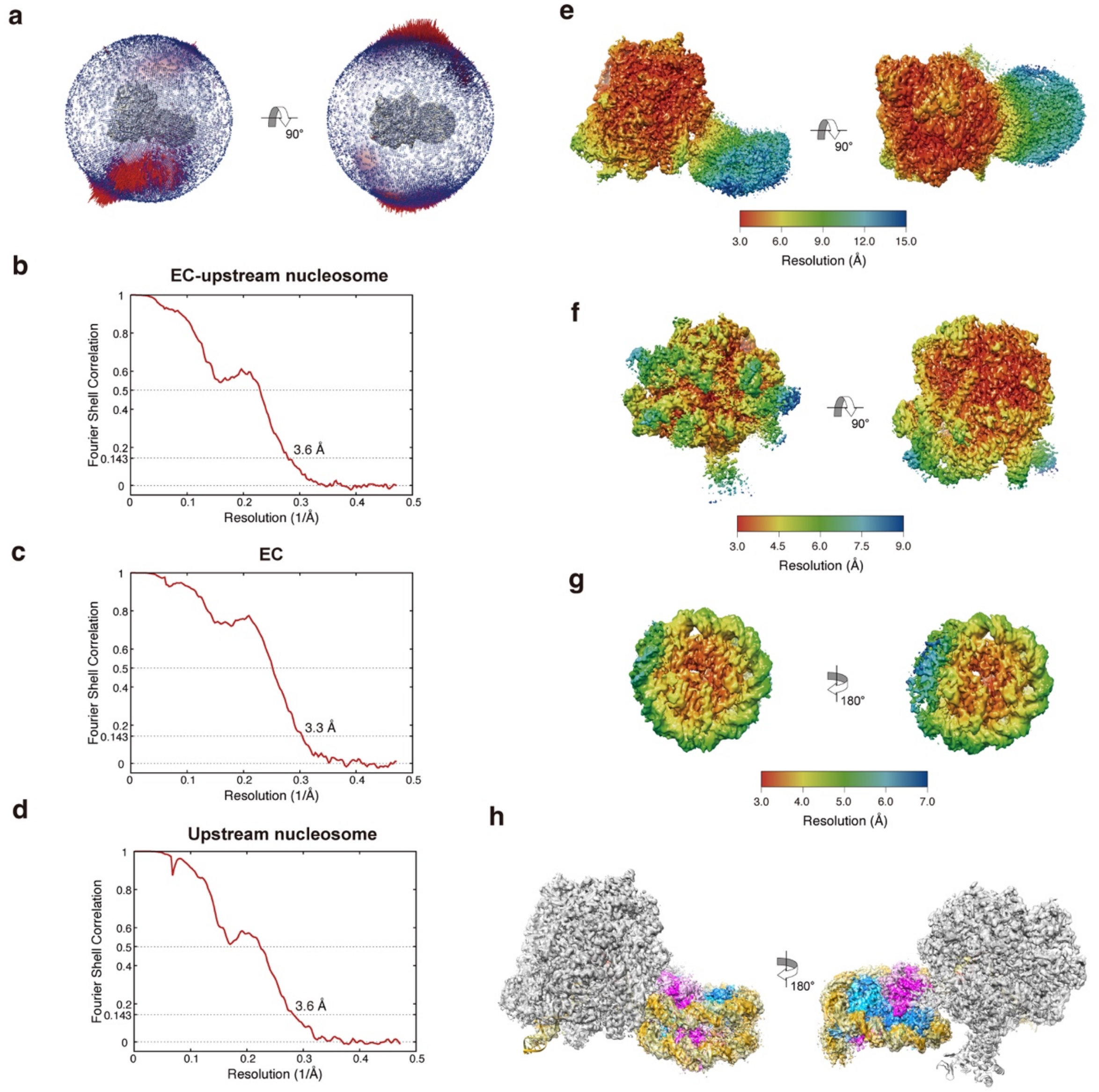

Supplementary Fig. 7. Quality of the EC-upstream nucleosome structure. (a) Euler angular distribution of the structure. **(b-d)** Fourier Shell Correlation (FSC) curves for the EC-upstream nucleosome (b), EC (c), and upstream nucleosome (d). The resolutions of the EC-upstream nucleosome, EC, and upstream nucleosome structures were estimated to be 3.6, 3.3, and 3.6 Å, respectively, by an FSC = 0.143. **(e-g)** Local resolution maps of the EC-upstream nucleosome (e), EC (f), and upstream nucleosome (g). **(h)** Cryo-EM density of the EC-upstream nucleosome composite map with the fitted atomic model.

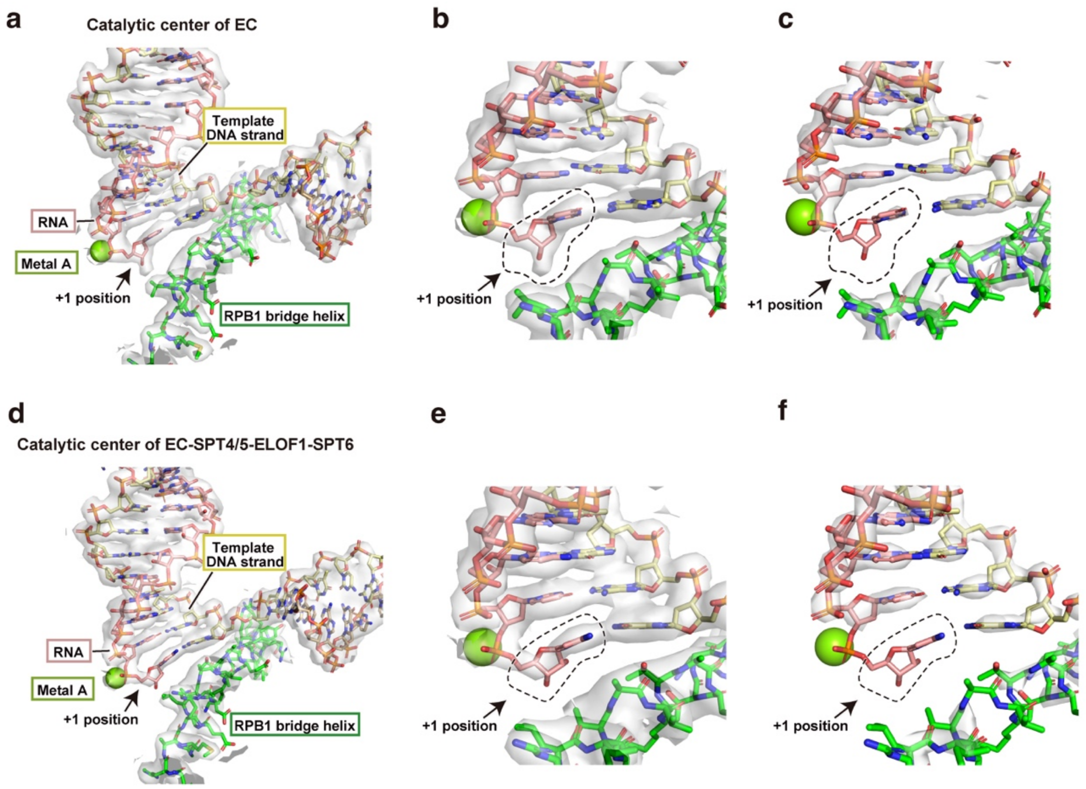

Supplementary Fig. 8. Catalytic centers of EC and EC-SPT4/5-ELOF1-SPT6. (a) The catalytic center of EC. **(b,c)** The +1 RNA base density at low (b) and high (c) contour levels of EC. **(d)** The catalytic center of EC-SPT4/5-ELOF1-SPT6. **(e,f)** The +1 RNA base density at low (e) and high (f) contour levels of EC-SPT4/5-ELOF1-SPT6. The DNA and RNA densities with the fitted atomic models are shown. The green sphere indicates the magnesium ion called metal A, which indicates the location of the catalytic center. The +1 position of the base in the catalytic center is indicated.

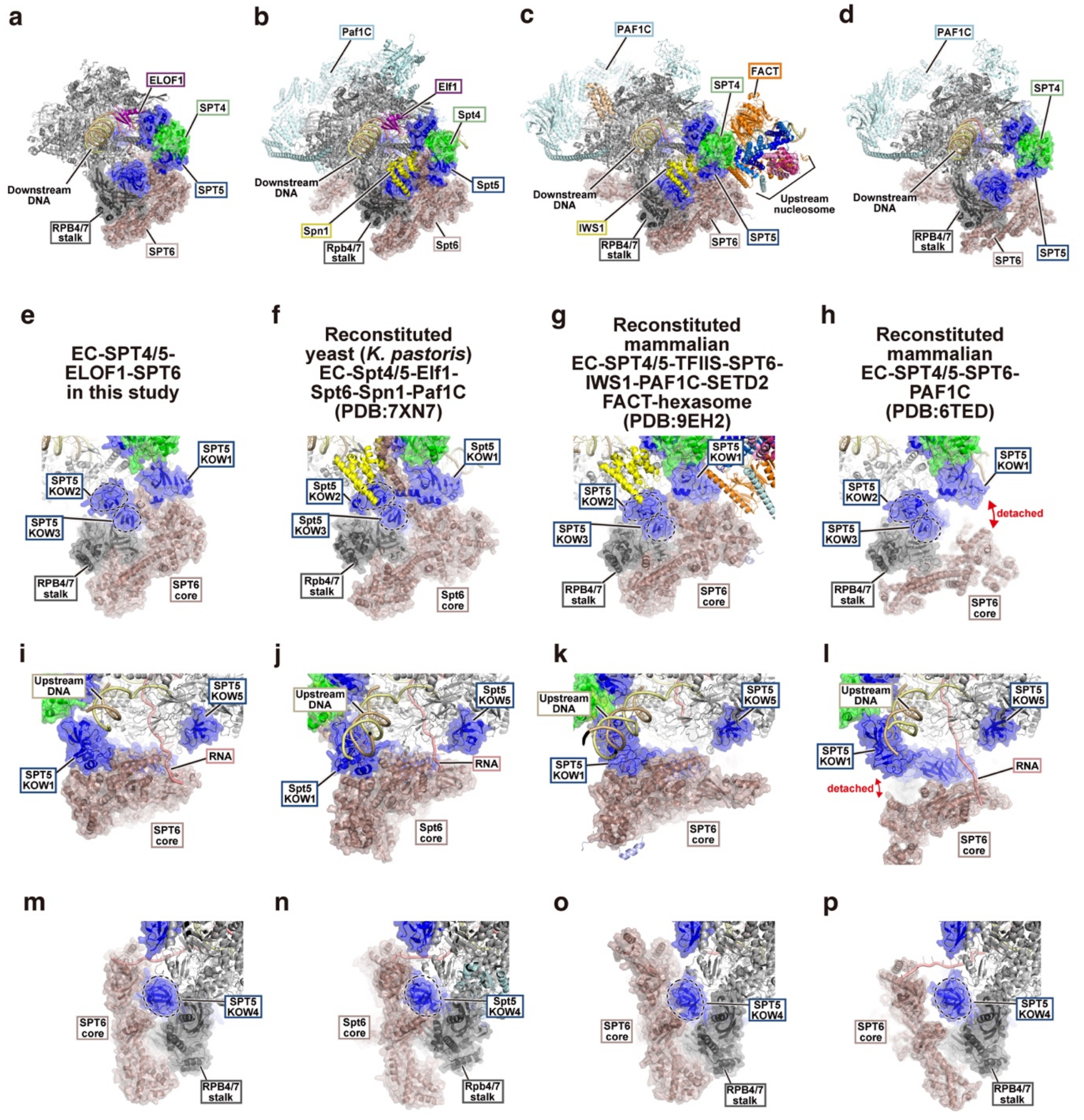

Supplementary Fig. 9. Structural comparison of ECs complexed with transcription elongation factors. (a,e,i, and m) Structure of the EC-SPT4/5-ELOF1-SPT6 complex in this study. **(b, f, j, and n)** Structure of the reconstituted yeast (*K. pastoris*) EC-Spt4/5-Elf1-Spt6-Spn1-Paf1C complex (PDB: 7XN7). **(c, g, k, and o)** Structure of the reconstituted mammalian EC-SPT4/5-TFIIS-SPT6-IWS1-PAF1C-SETD2-FACT-hexasome complex (PDB:9EH2). **(d, h, l, and p)** Structure of the reconstituted mammalian EC-SPT4/5- SPT6-PAF1C complex (PDB:6TED). **(e-h)** Close-up views of RPB4/7 stalk-SPT5 (KOW1 and 3)-SPT6 interaction. **(i-l)** Close-up views of SPT5 KOW1-SPT6 interaction. PAF1C and FACT-histone complexes are omitted for clarity. **(m-p)** Close-up views of SPT5 KOW4-SPT6 interaction.

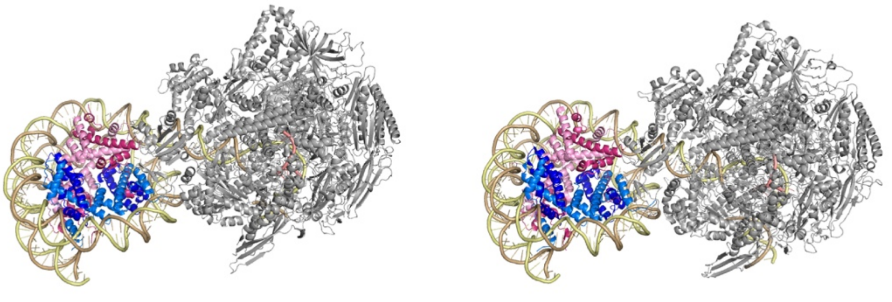

Supplementary Fig. 10. Comparison between the EC-downstream nucleosome and reconstituted yeast EC-nucleosome complexes. The structures of the EC-downstream nucleosome in this study and the reconstituted yeast (*K. pastoris*) EC-nucleosome, in which EC is paused at the SHL(-5) position in the nucleosome, are shown.

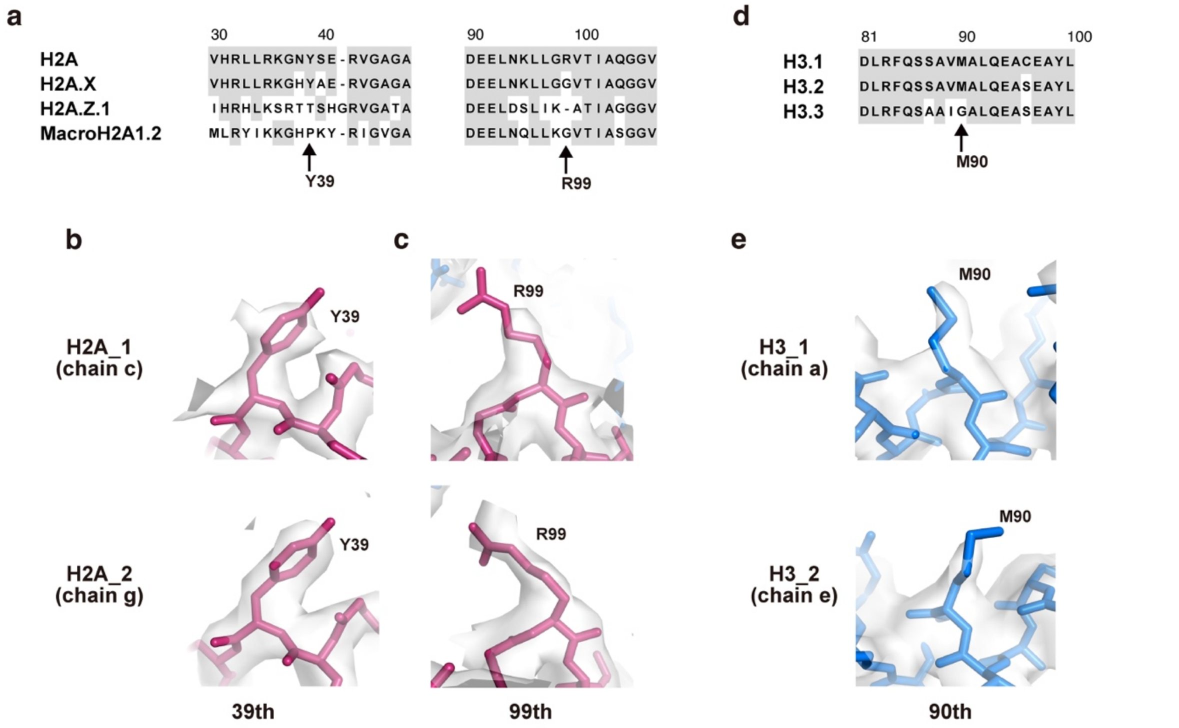

Supplementary Fig. 11. CryoEM density of the histone side chains in the EC-upstream nucleosome. (a) Amino acid sequence alignment of the histone H2A variants. **(b, c)** The side chain densities of the 39th (b) and 99th (c) residues of H2A. The two H2A molecules contained in the nucleosome are shown. **(d)** Amino acid sequence alignment of the histone H3 variants. **(e)** The side chain densities of the 90th residues of H3. The two H3 molecules contained in the nucleosome are shown.

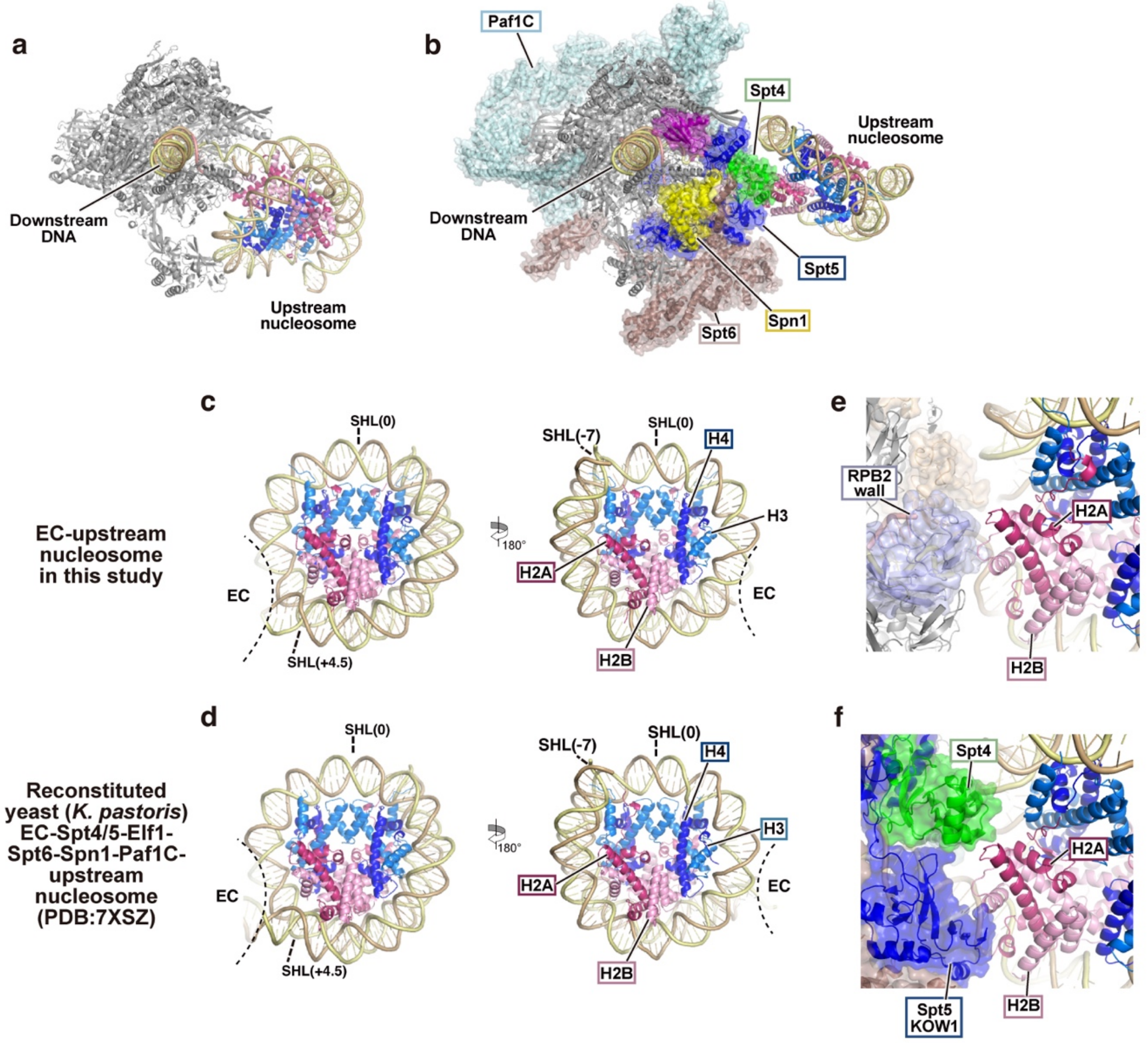

Supplementary Fig. 12. Comparison between the EC-upstream nucleosome and reconstituted yeast EC-Spt4/5-Elf1-Spt6-Spn1-Paf1C-upstream nucleosome (EC115) complexes. (a, b) The structures of the EC-upstream nucleosome in this study (a) and the EC-Spt4/5-Elf1-Spt6-Spn1-Paf1C-upstream nucleosome (EC115) complex (PDB: 7XSZ) (b). **(c,d)** Close-up views of the upstream nucleosome structures of the EC-upstream nucleosome (c) and EC115 (d). **(e)** Close-up view of the H2A-H2B dimer-RPB2 wall interaction. **(f)** Close-up view of the H2A-H2B dimer-Spt4-Spt5 KOW1 interaction.

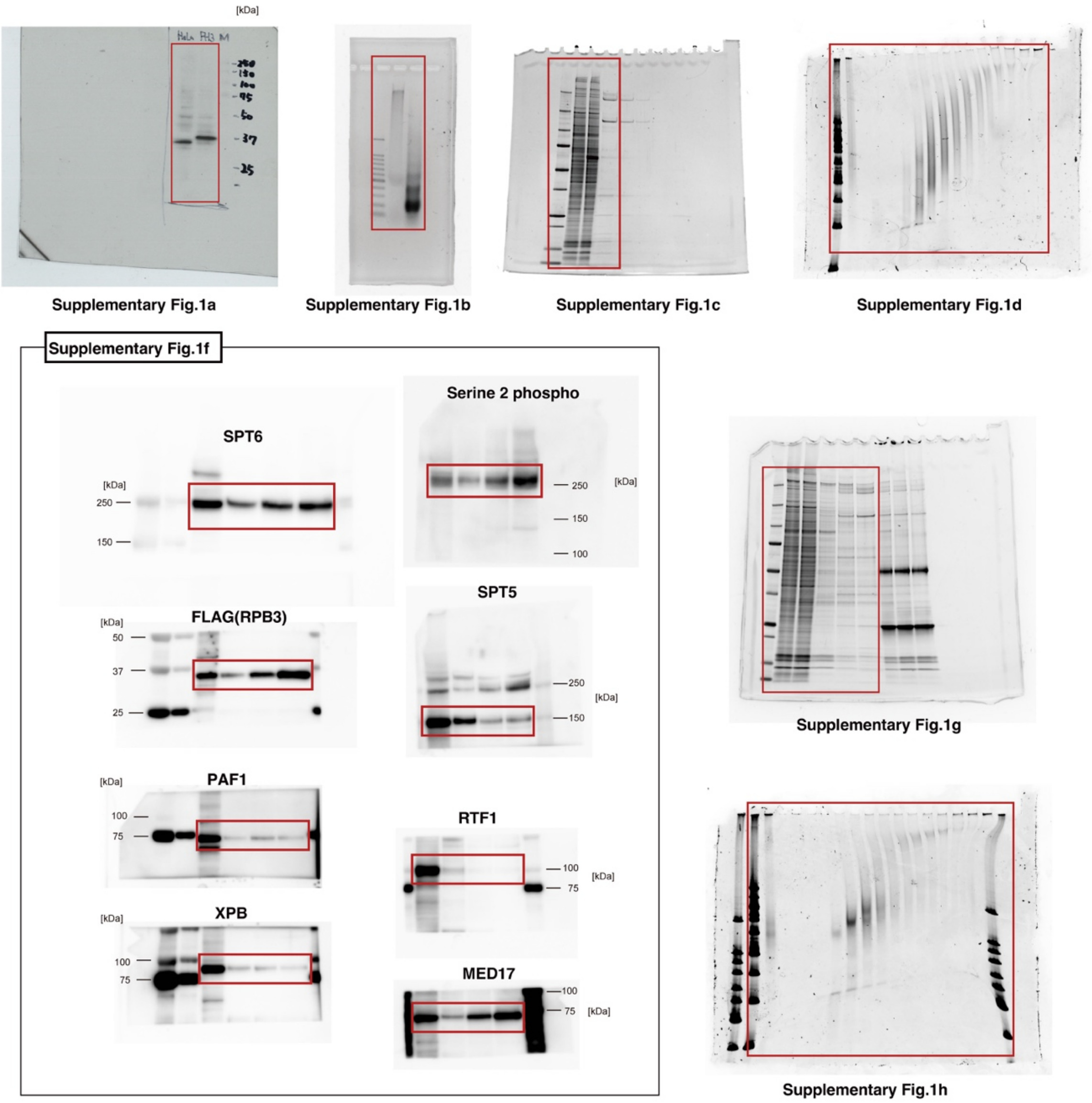

Source Data of Supplementary Fig. 1. The uncropped scans of all blots and gels. The areas shown in the Supplementary Fig. 1 are indicated by red rectangles.
