# Supplementary material for: Multiple structures of RNA polymerase II isolated from human nuclei by ChIP-CryoEM analysis
Source: Nat Commun. 2025 May 28;16:4724. doi: 10.1038/s41467-025-59580-x (PMC12119854; doi:10.1038/s41467-025-59580-x)
Supplement: Supplementary file 3 — Description of Additional Supplementary Files [file 41467_2025_59580_MOESM3_ESM.pdf]

Description of Additional Supplementary Files

File Name: Supplementary Data 1

Description: The RNAPII-associated proteins identified by LC-MS/MS analysis (full list)
