# Supplementary material for: Multiple structures of RNA polymerase II isolated from human nuclei by ChIP-CryoEM analysis
Source: Nat Commun. 2025 May 28;16:4724. doi: 10.1038/s41467-025-59580-x (PMC12119854; doi:10.1038/s41467-025-59580-x)
Supplement: Supplementary file 5 — Reporting Summary [file 41467_2025_59580_MOESM5_ESM.pdf]

nature portfolio

Corresponding author(s): Hitoshi Kurumizaka

Last updated by author(s): Mar 11, 2025

Reporting Summary

Nature Portfolio wishes to improve the reproducibility of the work that we publish. This form provides structure for consistency and transparency in reporting. For further information on Nature Portfolio policies, see our Editorial Policies and the Editorial Policy Checklist.

Statistics

For all statistical analyses, confirm that the following items are present in the figure legend, table legend, main text, or Methods section.

n/a	Confirmed	
☒	☐	The exact sample size (*n*) for each experimental group/condition, given as a discrete number and unit of measurement
☒	☐	A statement on whether measurements were taken from distinct samples or whether the same sample was measured repeatedly
☒	☐	The statistical test(s) used AND whether they are one- or two-sided<br>*Only common tests should be described solely by name; describe more complex techniques in the Methods section.*
☒	☐	A description of all covariates tested
☒	☐	A description of any assumptions or corrections, such as tests of normality and adjustment for multiple comparisons
☒	☐	A full description of the statistical parameters including central tendency (e.g. means) or other basic estimates (e.g. regression coefficient) AND variation (e.g. standard deviation) or associated estimates of uncertainty (e.g. confidence intervals)
☒	☐	For null hypothesis testing, the test statistic (e.g. *F*, *t*, *r*) with confidence intervals, effect sizes, degrees of freedom and *P* value noted<br>*Give P values as exact values whenever suitable.*
☒	☐	For Bayesian analysis, information on the choice of priors and Markov chain Monte Carlo settings
☒	☐	For hierarchical and complex designs, identification of the appropriate level for tests and full reporting of outcomes
☒	☐	Estimates of effect sizes (e.g. Cohen's *d*, Pearson's *r*), indicating how they were calculated

Our web collection on statistics for biologists contains articles on many of the points above.

Software and code

Policy information about availability of computer code

Data collection	EPU (ver.3.1 and 3.2, Thermo Fisher Scientific)
Data analysis	RELION (ver. 4.0), MotionCor2 (ver. 1.4.0), CTFFIND (ver. 4.1), UCSF Chimera (ver. 1.18), Phenix (ver.1.19.2 and 1.20.1), Coot (ver. 0.95), ISOLDE (ver. 1.3), UCSF ChimeraX (ver. 1.3), MolProbity (Phenix (ver.1.19.2 and 1.20.1)), PyMOL (ver. 2.5.5), Proteome Discoverer (version 2.4)

For manuscripts utilizing custom algorithms or software that are central to the research but not yet described in published literature, software must be made available to editors and reviewers. We strongly encourage code deposition in a community repository (e.g. GitHub). See the Nature Portfolio guidelines for submitting code & software for further information.

Data

Policy information about availability of data

All manuscripts must include a data availability statement. This statement should provide the following information, where applicable:

- Accession codes, unique identifiers, or web links for publicly available datasets
- A description of any restrictions on data availability
- For clinical datasets or third party data, please ensure that the statement adheres to our policy

The cryo-EM reconstructions and atomic models of the RNAPII EC have been deposited in the Electron Microscopy Data Bank and the Protein Data Bank (PDB) under the accession codes: EC: PDB ID 8XSO and EMDB entry ID EMD-38624; EC-SPT4/5-ELOF1-SPT6 : PDB ID 8XRM and EMDB entry ID EMD-38607; EC-

downstream nucleosome: PDB ID 8XVS and EMDB entry ID EMD-38717; EC-upstream nucleosome : PDB ID 8XRJ and EMDB entry ID EMD-38604. The raw mass spectrometry data used in this study have been deposited to the proteomeXchange Consortium (PXD052434) via the Japan ProteOme STandard (JPOST) repository under the accession code : JPST003120.

Research involving human participants, their data, or biological material

Policy information about studies with human participants or human data. See also policy information about sex, gender (identity/presentation), and sexual orientation and race, ethnicity and racism.

Reporting on sex and gender	N/A
Reporting on race, ethnicity, or other socially relevant groupings	N/A
Population characteristics	N/A
Recruitment	N/A
Ethics oversight	N/A

Note that full information on the approval of the study protocol must also be provided in the manuscript.

Field-specific reporting

Please select the one below that is the best fit for your research. If you are not sure, read the appropriate sections before making your selection.

[x] Life sciences [ ] Behavioural & social sciences [ ] Ecological, evolutionary & environmental sciences

For a reference copy of the document with all sections, see nature.com/documents/nr-reporting-summary-flat.pdf

Life sciences study design

All studies must disclose on these points even when the disclosure is negative.

Sample size	For the biochemical experiments, sample sizes were not calculated because no statistical analysis was performed. 32,975 micrographs were used in the cryoEM anlaysis, which are sufficient for structual reconstitution for the discussion, as shown in the Supplementary figures.
Data exclusions	During cryo-EM analyses, bad particles were excluded as shown in Supplementary Figures. Other than the cryo-EM analyses, no data was excluded.
Replication	The reproducibility of the findings were confirmed by performing at least two independent experiments. Cryo-EM analysis was conducted once because the final map already represented the average of a large number of images.
Randomization	Data was not randomized during biochemical analyses. Cryo-EM processing software Relion randomly splits particles into two different half-maps during 3D reconstruction in order to calculate resolution of the 3D volume. Randomization was not relevant to biochemical experiments.
Blinding	Since there was no subjective allocation in our investigation, blinding was irrelevant to this study.

Reporting for specific materials, systems and methods

We require information from authors about some types of materials, experimental systems and methods used in many studies. Here, indicate whether each material, system or method listed is relevant to your study. If you are not sure if a list item applies to your research, read the appropriate section before selecting a response.

Materials & experimental systems

n/a	Involved in the study	
[ ]	[x]	Antibodies
[ ]	[x]	Eukaryotic cell lines
[x]	[ ]	Palaeontology and archaeology
[x]	[ ]	Animals and other organisms
[x]	[ ]	Clinical data
[x]	[ ]	Dual use research of concern
[x]	[ ]	Plants

Methods

n/a	Involved in the study	
[x]	[ ]	ChIP-seq
[x]	[ ]	Flow cytometry
[x]	[ ]	MRI-based neuroimaging

Antibodies

Antibodies used

1. anti-RPB3 (Bethyl Cat#A303-771, 1:6,000)
2. anti-FLAG M2 antibody (Sigma # F3165, 1:3,000)
3. anti-SPT5 antibody (Proteintech#16511-1-AP, 1:1,000)
4. anti-SPT5 antibody (Cell Signalling #9033S, 1:1,000)
5. anti-SPT6 antibody (Cell Signalling #15616, 1:2,000)
6. anti-PAF1 antibody (Cell Signalling #12883, 1:1,000)
7. anti-RTF1 antibody (Proteintech #12170-1-AP, 1:1,000)
8. anti-MED17 antibody (Proteintech#11505-1-AP, 1:2,000)
9. anti-XPB antibody (Cell Signalling #8746, 1:300)

Validation

1. Western blot of various cell lines was performed. (https://www.biomol.com/products/antibodies/primary-antibodies/general/anti-rpb3-a303-771a-t#)
2. The FLAG M2 antibody has been validated by western blot of various cell lines was performed.(https://www.sigmaaldrich.com/JP/ja/product/sigma/b3111)
3. Western blot of various cell lines was performed. (https://www.ptglab.com/products/SUPT5H-Antibody-16511-1-AP.htm?srsltid=AfmBOoqvEMA26ybKLNvA-8zq7Jb6xZHz2V9wpNRHCnWYTR2lBsJP8T5N)
4. Western blot of various cell lines was performed. (https://www.cellsignal.jp/products/primary-antibodies/spt5-antibody/9033)
5. Western blot of various cell lines was performed. (https://www.cellsignal.com/products/primary-antibodies/spt6-d6j9h-rabbit-mab/15616?srsltid=AfmBOopwh-xDI0tudEoIpWxiAj0R_4JzluUEDyRsYC3VW7Ko5S3zaPsp)
6. Western blot of various cell lines was performed.(https://www.cellsignal.com/products/primary-antibodies/paf1-d9g9x-rabbit-mab/12883?srsltid=AfmBOoq_7Yo5x2HZylT2gYkfiDr52TZ9VGnQPZf_5JUIT7PT93A45AUW)
7. Western blot of various cell lines was performed. (https://www.ptglab.com/products/RTF1-Antibody-12170-1-AP.htm?srsltid=AfmBOopXP1JiqOnrlypCLS66qeDQIZczI9T1bWrKPzRH051onYQvNkka)
8. Western blot of various cell lines was performed. (https://www.ptglab.co.jp/products/MED17-Antibody-11505-1-AP.htm)
9. Western blot of various cell lines was performed.(https://awsqa-www.cellsignal.com/products/primary-antibodies/xpb-2c6-mouse-mab/8746)

Eukaryotic cell lines

Policy information about cell lines and Sex and Gender in Research

Cell line source(s)

HeLa cells expressing FLAG-His-RPB3 established in Hasegawa et al., J. Biochem, 2003.

Authentication

The cell line was was authenticated by western blot by detecting FLAG-tagged RPB3 and STR analysis.

Mycoplasma contamination

The cell line is tested negative for the Mycoplasma contamination.

Commonly misidentified lines
(See ICLAC register)

No commonly misidentified cell lines were used in the study.

Plants

Seed stocks

N/A

Novel plant genotypes

N/A

Authentication

N/A
