# Supplementary figures and images for: Multiple structures of RNA polymerase II isolated from human nuclei by ChIP-CryoEM analysis
Source: Nat Commun. 2025 May 28;16:4724. doi: 10.1038/s41467-025-59580-x (PMC12119854; doi:10.1038/s41467-025-59580-x)

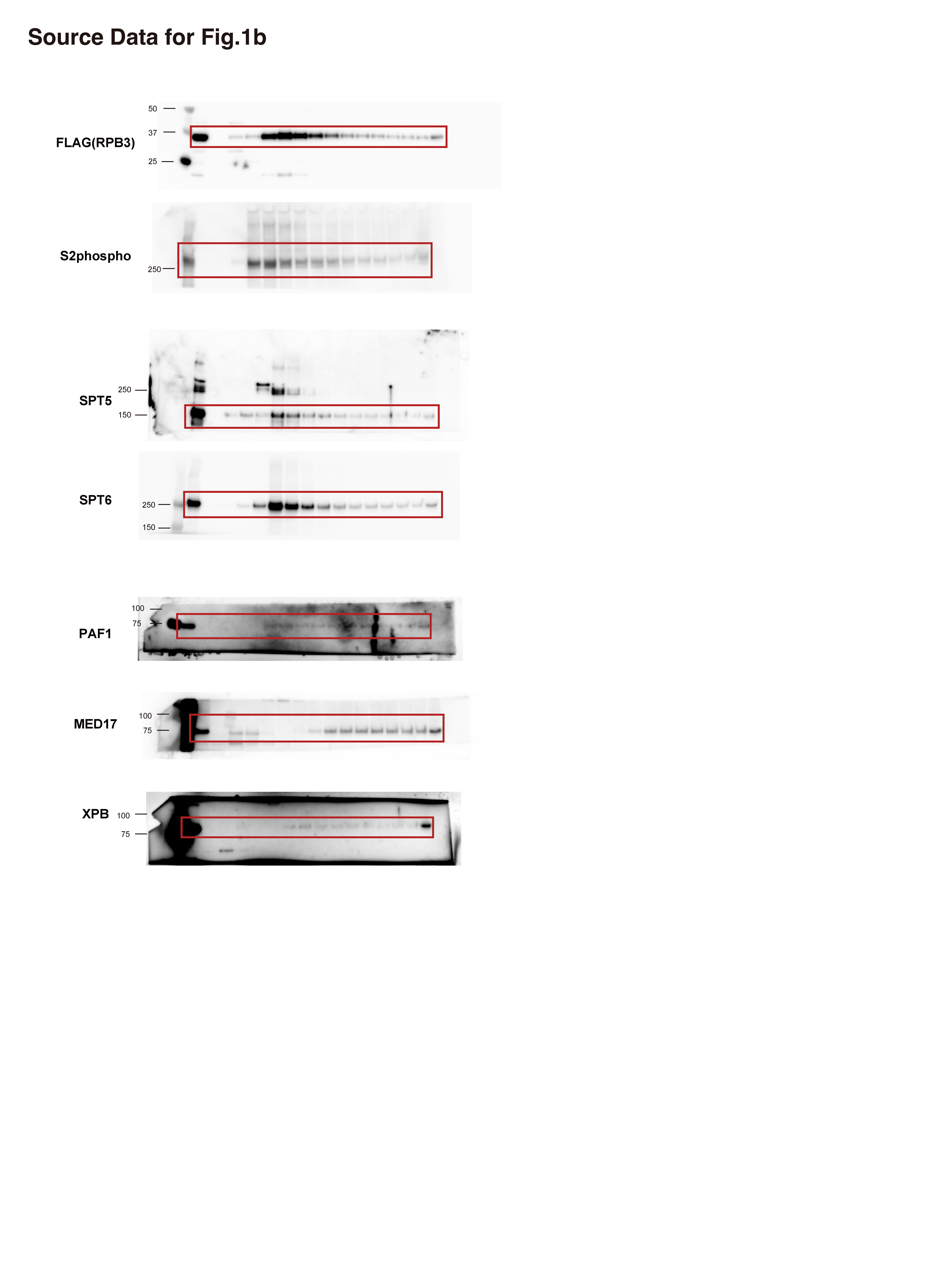

Supplement: Supplementary file 6 — Source Data [file 41467_2025_59580_MOESM6_ESM.zip › Source Data/Kujirai_SD_Fig1.jpg]
